# The impact of salient action effects on 6-, 7-, and 11-month-olds’ goal-predictive gaze shifts for a human grasping action

**DOI:** 10.1371/journal.pone.0240165

**Published:** 2020-10-02

**Authors:** Maurits Adam, Birgit Elsner

**Affiliations:** Department of Psychology, University of Potsdam, Potsdam, Brandenburg, Germany; Pontificia Universidad Catolica de Chile, CHILE

## Abstract

When infants observe a human grasping action, experience-based accounts predict that all infants familiar with grasping actions should be able to predict the goal regardless of additional agency cues such as an action effect. Cue-based accounts, however, suggest that infants use agency cues to identify and predict action goals when the action or the agent is not familiar. From these accounts, we hypothesized that younger infants would need additional agency cues such as a salient action effect to predict the goal of a human grasping action, whereas older infants should be able to predict the goal regardless of agency cues. In three experiments, we presented 6-, 7-, and 11-month-olds with videos of a manual grasping action presented either with or without an additional salient action effect (Exp. 1 and 2), or we presented 7-month-olds with videos of a mechanical claw performing a grasping action presented with a salient action effect (Exp. 3). The 6-month-olds showed tracking gaze behavior, and the 11-month-olds showed predictive gaze behavior, regardless of the action effect. However, the 7-month-olds showed predictive gaze behavior in the action-effect condition, but tracking gaze behavior in the no-action-effect condition and in the action-effect condition with a mechanical claw. The results therefore support the idea that salient action effects are especially important for infants’ goal predictions from 7 months on, and that this facilitating influence of action effects is selective for the observation of human hands.

## Introduction

When observing their own or others’ goal-directed actions, humans tend to look at the goal object before the manipulating agent arrives there, therefore predicting the action outcome or the final position of the action [1, 2]. This action-goal prediction is suggested to be important for successful interaction with the environment because it helps us to keep up with the speed of actions and action goals while they unfold [3]. Infants show predictive gaze behavior, that is, gaze shifts to the goal object before the action’s end state is achieved, for simple actions already in the first year of life. For example, 11-month-olds successfully predicted the action goal of a hand putting toys into a container [1], and 6- and 10-month-olds predicted the final position (i.e., the mouth region) of manual feeding actions with a spoon [4]. However, infants’ ability to predict the goal or the final position of an ongoing action seems to be closely linked to the familiarity of the action and the agent, because infants often fail to show predictive gaze behavior for unfamiliar actions, such as back-of-hand touching, or for unfamiliar agents, such as mechanical claws [5, 6]. Thus, the main goal of the present study was to examine critical factors that influence infants’ goal-predictive gaze behavior in the first year of life.

Some researchers have suggested that during action observation, the perceived motor information is mapped onto own motor representations of that action, and this direct matching process then enables the observer to predict the action goal [1, 7]. This experience-based theory serves as an explanation for infants’ failure to predict the goals of unfamiliar actions or agents, where the corresponding motor information is not available. A cue-based approach, however, suggests that infants also use certain contextual and behavioral cues to process observed actions and predict action goals. Indeed, looking-times of 6-, 9-, and 12-month-olds indicated that the infants processed the action-goal when an unfamiliar agent (e.g., a grasping mechanical claw) exhibited agency cues such as self-propelled movement, equifinality of goal achievement, or a salient action effect [8]. Supposedly, agency cues trigger an innate core notion of goal-directed agents that in turn enables infants to process the goal-directedness of an observed action [9, 10]. Over time, with repeated exposure to goal-directed actions, infants will learn, for example, how agents such as hands or mechanical claws look, move, and typically manipulate goal objects. Eventually, when infants see how a hand or a claw approaches a goal object, the infants’ memory about the associated properties will be triggered even in the absence of visible agency cues, which in turn leads to the detection of goal-directedness or the production of action-goal predictions [8, 11, 12]. Influences of agency cues on goal-related looking-times—measured after completion of the action—have been found already from 5 months onwards [8, 13–15], and younger infants apparently need to see more agency cues than older infants in order to form an expectation about the to-be-achieved action goal [8].

The present study investigated infants of different ages, because systematic investigations on the influences of agency cues on action-goal predictions—measured online as the action unfolds—are still scarce. Such influences have been reported in eye-tracking studies for 13-month-olds [16] and 11-month-olds [17, 18]. Furthermore, studies on 11-month-olds’ goal-predictive gaze behavior indicate that agency cues are rendered redundant with increasing action experience, as suggested by the cue-based accounts [8, 11]. Around 11 months of age, infants are experienced graspers and showed predictive gaze behavior towards the goals of human grasping actions even when these actions followed a straight trajectory, did not show explicit cues for self-propelledness, and resulted only in touching of the goal object [6, 19]. Mere touching may not qualify as a salient action effect, because the term action effect usually refers to a change in the environment that was brought about by an agent [8, 13, 14]. However, for similar grasping actions of a mechanical claw, 11-month-olds showed goal-predictive gaze behavior only when these actions were accompanied by agency cues, or at least by the production of a salient action effect [17, 18]. In terms of the cue-based account [8, 11], this could mean that at 11 months, agency cues have been internalized to feature-specific aspects of the familiar human hand, but not to an unfamiliar mechanical claw. Additionally, 11-month-olds predicted the claw’s action goal equally fast when it was presented with all three cues mentioned above or with only one agency cue in the form of a salient action effect [17], suggesting that action effects play a specific role in goal prediction [12, 20].

In the light of these results, it seems possible that infants have to draw on potentially available agency cues—especially salient action effects [17]—to predict an action goal when an agent or action is less familiar, but can draw on their knowledge about the observed action and the outcome it is going to produce when an agent or action is highly familiar [11–13, 18]. The present study examined whether older infants would be able to predict the goal of a grasping human hand without any additional agency cues, probably based on their richer active or observational experience with human goal-directed grasping [8, 13]. Seeing the familiar agent and the start of the familiar reaching movement could trigger forward modeling, based on the stored action representation, enabling action-goal prediction via top-down influences [12]. In contrast, when younger infants observe the same grasping action, they might benefit from the addition of agency cues such as a salient action effect (bottom-up action information), because they have not gathered sufficient experience or action knowledge to predict the action goal from the visible agent features and movement cues alone [12]. Therefore, the present eye-tracking study investigated different age groups and their need to draw on agency cues when predicting the goals of observed actions in order to yield new insights into the interplay between external, bottom-up action-related features, and internal, top-down action knowledge.

In particular, the present study investigated the impact of a specific agency cue–the production of a salient action effect—on the occurrence of predictive goal-directed gaze behavior in three infant age groups (i.e., 6-, 7-, and 11-month-olds). Based on prior looking-time research, the salient action effect was operationalized as the agent bringing about a salient change of state in the goal object [8, 13, 14], that is, the agent lifted up the goal object, accompanied by an interesting sound, and then put the object back in its original position. Our main questions were first, at which age infants need to draw on salient action effects to visually predict the goal of an ongoing human grasping action, second, whether production of a salient action effect triggers goal-predictive gaze behavior also for an identical action of an unfamiliar agent (i.e., mechanical claw), and third, whether the infants would show a learning progress across trials, indicated by faster predictions across the course of the experiment. Based on prior research, we expected predictive gaze shifts to reflect gaze behavior as if the observer would perform the action themselves. In the context of grasping actions, this means gaze shifts towards the goal object [17, 18], and not towards the peak of the lifting movement, because predictive looks during own grasping would also mostly be aimed towards the to-be grasped object [2].

In Experiment 1, we presented 6- and 11-month-olds with videos of a human grasping action, either with or without a salient action effect. At 6 months, infants already have some experience with visually-guided grasping (starting from about 5 months), however, their grasp control when reaching for and grasping a goal object is still based on tactile feedback [21]. This may explain why 6-month-olds can detect a (mis)match between an expected and an achieved human action goal in looking-time studies, after having received complete action information, followed by a salient action effect [13, 14]. However, for 6-month-olds’ goal prediction for ongoing human grasping actions that do not display obvious agency cues, findings are inconsistent. Some studies reported goal-predictive gaze behavior [6, 22], others did not [1, 23]. Therefore, an action-related agency cue such as a salient action effect might help 6-month-olds to activate their knowledge about human grasping, enabling them to shift their gaze predictively from the familiar moving agent towards the goal object [12]. In contrast, at 11 months, infants’ rich experience with own and observed human grasping should enable action-goal prediction from seeing the familiar agent and the start of a familiar reaching movement via top-down influences, based on stored action representations [12], such that the agency cue in form of a salient action effect should be rendered irrelevant [8].

Because the 6-month-olds in Experiment 1 did not show predictive gaze behavior, we presented 7-month-olds in Experiment 2 with the same videos as in Experiment 1 to test whether infants with more knowledge about human grasping would be able to draw on the agency cue of a salient action effect to generate goal-predictive gaze behavior. Around 7 months of age, infants change from a mainly tactile feedback-based to a more visually-based and prospective grasp control during active reaching and grasping of a goal object [21]. Therefore, perceptual information about produced action effects might enable 7-month-olds to activate their stored action representations during the first few observations of a human grasping action, enabling online prediction of the upcoming action goal via top-down influences in the subsequent trials [12]. However, if the infants’ ability to produce predictive gaze shifts is based on experience with own or observed actions, it should be restricted to human grasping. In Experiment 3, we therefore presented 7-month-olds with videos of a mechanical claw performing a grasping action with a salient action effect, to rule out that 7-month-olds’ ability to draw on salient action effects is based on mere associative learning processes driven by general cognitive maturation.

## Experiment 1

Based on the idea of the interplay between bottom-up information (such as agent and movement features) and agency cues, and top-down information in the form of action knowledge [12], we expected 6-month-olds to show significantly faster gaze shifts towards the goal object of the observed human grasping action when a salient action effect was presented than when no action effect was presented. In particular, we expected the 6-month-olds to show predictive gaze behavior in the action-effect condition, but not in the no-action-effect condition. Furthermore, we expected the 11-month-olds to show equally fast predictive gaze shifts both in the presence and the absence of a salient action effect. Regarding learning progress across trials, based on prior research, we expected the 6-month-olds to show faster gaze shifts across trials only in the presence of a salient action effect, and the 11-month-olds to show faster gaze shifts across trials in both the action-effect and no-action-effect condition [5, 18, 19].

### Method

#### Participants

The final sample consisted of fifty-one 6-month-olds (*M* = 6.1 months, *SD* = 0.3, range = 5.5–6.9 months; 23 girls) and fifty-two 11-month-olds (*M* = 11.4 months, *SD* = 0.3, range = 11.0–12.0 months; 28 girls). An additional twenty 6-month-olds and four 11-month-olds had to be excluded because they did not fulfill the inclusion criteria (see below). The number of infants tested in our experiments exceeded that of most prior research in this field. This ensures that our critical analyses regarding the threshold of 0 ms (see below) could be conducted with high statistical power to reveal predictive gaze behavior. We calculated post-hoc power analyses using G-Power based on the conducted *t*-tests with alpha = .05 and estimated big effect sizes between 0.89–1.54 in the context of predictive gaze behavior, and this revealed a statistical power of = 1.0 for our analyses. The infants were randomly assigned to the action-effect condition (*n* = 26 per age group) or the no-action-effect condition (*n* = 25–26 per age group). The parents and their infants were invited by phone from a database with infants from a medium-sized German city. The participants were predominantly German and came from middle-class families. The database consisted of data from families who had voluntarily signed up via phone or mail and agreed to participate in studies conducted in the babylab. Upon arrival at the lab, the parents signed informed consent, and as reimbursement for their expenses, they received 7.50 € as well as a certificate with a photo of their infant. This research was approved by the Ethics committee of the University of Potsdam based on Ethics application Nr. 49/2017.

#### Stimuli and apparatus

**Fig 1:**
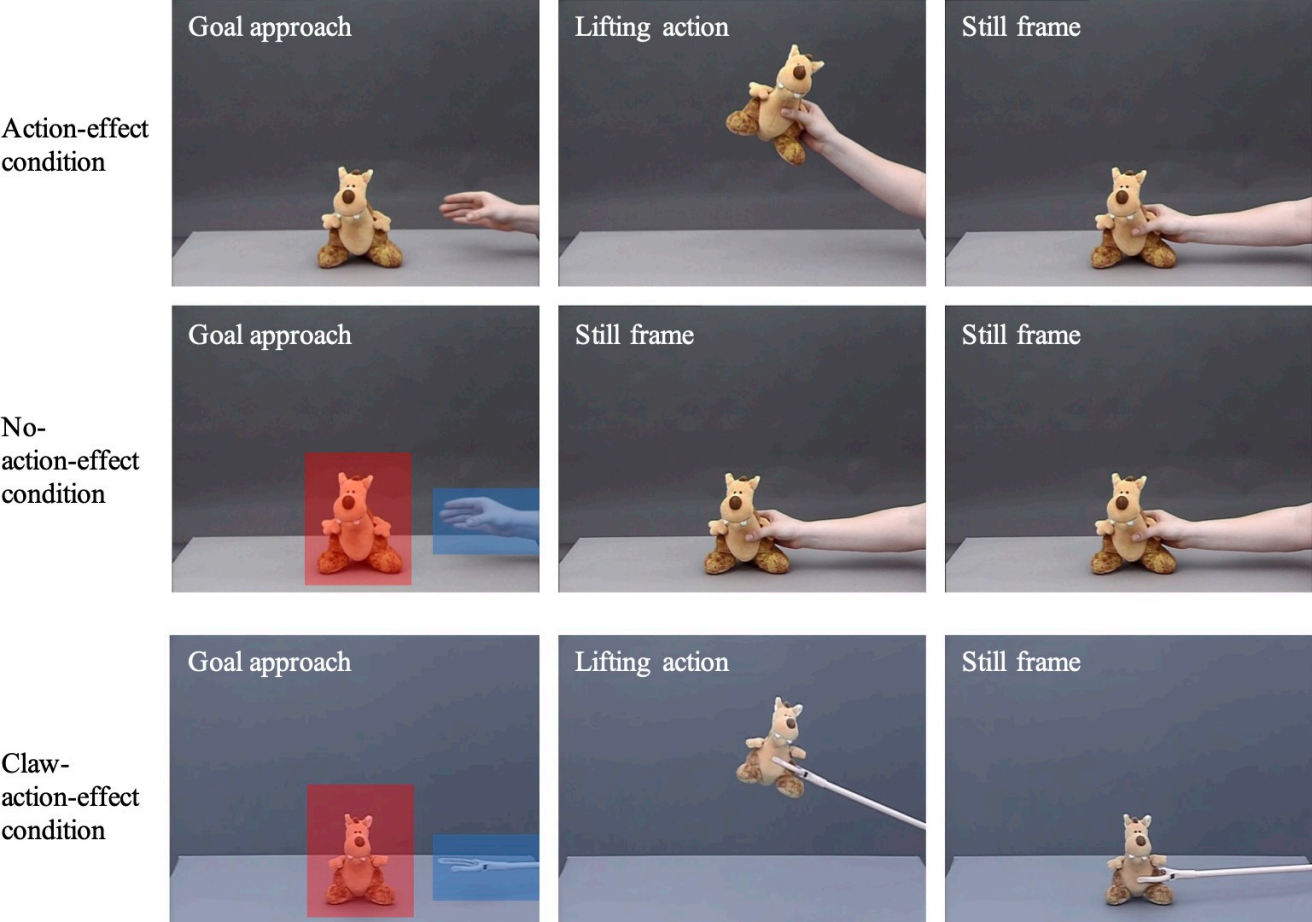
Still frames of the stimulus videos taken from the action-effect condition (upper row) and the no-action-effect condition (middle row) of Experiment 1 and 2 as well as from the claw-action-effect condition (lower row) of Experiment 3. The squares in the left pictures of the no-action-effect condition and the claw-action-effect condition represent examples of the AOIs used for data analysis (the squares were not visible during the experiment).

The experiment consisted of a grasping video that was presented 12 times. Video stills of the stimuli for the action-effect- and no-action-effect condition are depicted in Fig 1. In both conditions, a toy animal sat at screen center on a gray table in front of a gray background. After 1000 ms, a hand entered the scene from the right and approached the toy on a linear path (movement duration: approx. 2500 ms). Infants’ gaze behavior was analyzed during goal approach, until the hand had reached the toy, and until that point, the videos in both conditions were identical. In the action-effect condition, the hand then lifted up the toy with an accompanying sound and put the toy back on the table (action duration: approx. 2800 ms), followed by a still frame (duration: approx. 2400 ms). In the no-action-effect condition, the hand froze in place as soon as it had grasped the toy and stayed in this position for approx. 5200 ms. Therefore, the duration and speed of the initial grasping movement as well as the total duration of the videos (approx. 8700 ms) were the same in both conditions.

Participants’ gaze behavior was recorded during the goal approach with an SMI REDm eye tracker with a sampling rate of 250 Hz mounted to a 22-inch monitor with a screen resolution of 1680 x 1050 pixels. Before the experiment started, a 5-point calibration with an animated, pulsating ball in front of a gray background was used. Calibration points were accepted manually with fast calibration speed. Between trials, an attention getter (e.g., a jumping clock or a waving hand with an accompanying sound) was presented.

#### Procedure

The infants sat on their parents’ laps in front of the monitor at a distance of approximately 60 cm. Parents were instructed to not interact with their infant during the experiment. Following a successful 5-point-calibration procedure, the grasping videos with or without the action effect (action effect-condition vs. no-action-effect condition) were presented 12 times, and the infant’s gaze behavior was recorded in every one of these 12 trials. The infants and their parents spent approximately 20 minutes in the babylab, with an experiment run-time of about 4 minutes.

#### Data handling

To analyze gaze behavior, we created a moving AOI for the hand and a static AOI for the goal object (Fig 1). The same AOIs were used for both conditions.

Gaze-arrival times were calculated by subtracting the time of the infants’ first fixation of the goal AOI from the time when the hand entered the goal AOI. The first fixation was defined as first look at the goal AOI after the infant had fixated the moving hand AOI for at least 200 ms. Therefore, positive values indicate goal fixations before the hand reached the goal (predictive gaze shift), negative values indicate goal fixations after the hand reached the goal (reactive gaze shift), and a value of 0 ms indicates that the infant’s gaze and the hand entered the goal AOI at the same time (tracking gaze behavior). According to the conventions set in previous studies [1, 5, 6, 18, 19], a trial was considered valid when the infant first fixated the moving hand for at least 200 ms and then shifted their gaze to the goal object. Trials in which the infants’ gaze arrived at the goal AOI at least 1000 ms after the agent had entered the goal AOI were considered invalid. The first trial was excluded from the analyses, because the (non-)occurrence of the action effect happened only after gaze-arrival time measurement. From the remaining data from trials 2–12, infants needed at least 2 valid trials to be included in the analysis, a criterion previously used in eye-tracking studies with very young infants at the age of 6 months [6, 23]. The tracking ratio of the 6-month-olds in the action-effect condition (*M* = 69%, *SD* = 14) was significantly higher than in the no-action-effect condition (*M* = 56%, *SD* = 18), *t*(49) = -2.9, *p* < .01, *r* = .4. However, the 6-month-olds contributed a comparable number of valid trials in the action effect-condition (*M* = 5.8, *SD* = 3.1) and the no-action-effect condition (*M* = 6.7, *SD* = 2.5), *t*(49) = 1.1, *p* = .26, *r* = .1. The tracking ratio of the 11-month-olds in the action-effect condition (*M* = 90%, *SD* = 7) was also significantly higher than in the no-action-effect condition (*M* = 64%, *SD* = 18), *t*(50) = -6.6, *p* < .001, *r* = .7, and the 11-month-olds contributed significantly more valid trials in the action-effect condition (*M* = 9.0, *SD* = 1.7) than in the no-action-effect condition (*M* = 7.3, *SD* = 2.6), *t*(50) = -2.8, *p* < .01, *r* = .4. However, there was no significant correlation between the 11-month-olds’ number of valid trials and their mean gaze-arrival times, *r* = -.143, *p* = .313. Therefore, although the infants’ attention tended to be higher in the action-effect condition compared to the no-action-effect condition, probably due to the salient effect, this did not seem to have any systematic influence on the main variables.

To test our hypotheses, we used ANOVAs and independent samples *t*-tests to compare mean gaze-arrival time as a function of the two between-subjects factors age group and condition. For our critical analyses of the quality of infants’ gaze behavior, we used one-sample *t*-tests comparing the mean gaze-arrival times in each condition against the value of 0 ms, classifying gaze behavior as predictive, reactive, or tracking (see previous paragraph). Because some of our hypotheses regarding these *t*-tests predicted null-findings, we also included Bayes-Factors *BF*_01_ in favor of the null-hypothesis in these cases. Furthermore, we performed linear and curvilinear regression analyses on the mean gaze-arrival times across trials 2–12 for each age group and condition to investigate potential learning progress. For this analysis, we calculated single trial-means by averaging all valid data points in each trial of infants in a specific subgroup (e.g., the 6-month-olds in the action-effect condition). These single trial-means were then entered into a regression analysis with curve-fitting to test whether the changes in mean gaze-arrival times across trials 2–12 could be best described by a linear, quadratic, or logarithmic function. Finally, we used ANOVAs and *t*-tests to analyze mean looking-times to the agent and the goal object during the still frame and the goal approach (combined duration approx. 3500 ms) to rule out the possibility that the infants attended more to the action-effect condition compared to the no-action-effect condition, which might have made it easier to process the ongoing action in the action effect-condition.

### Results

**Fig 2:**
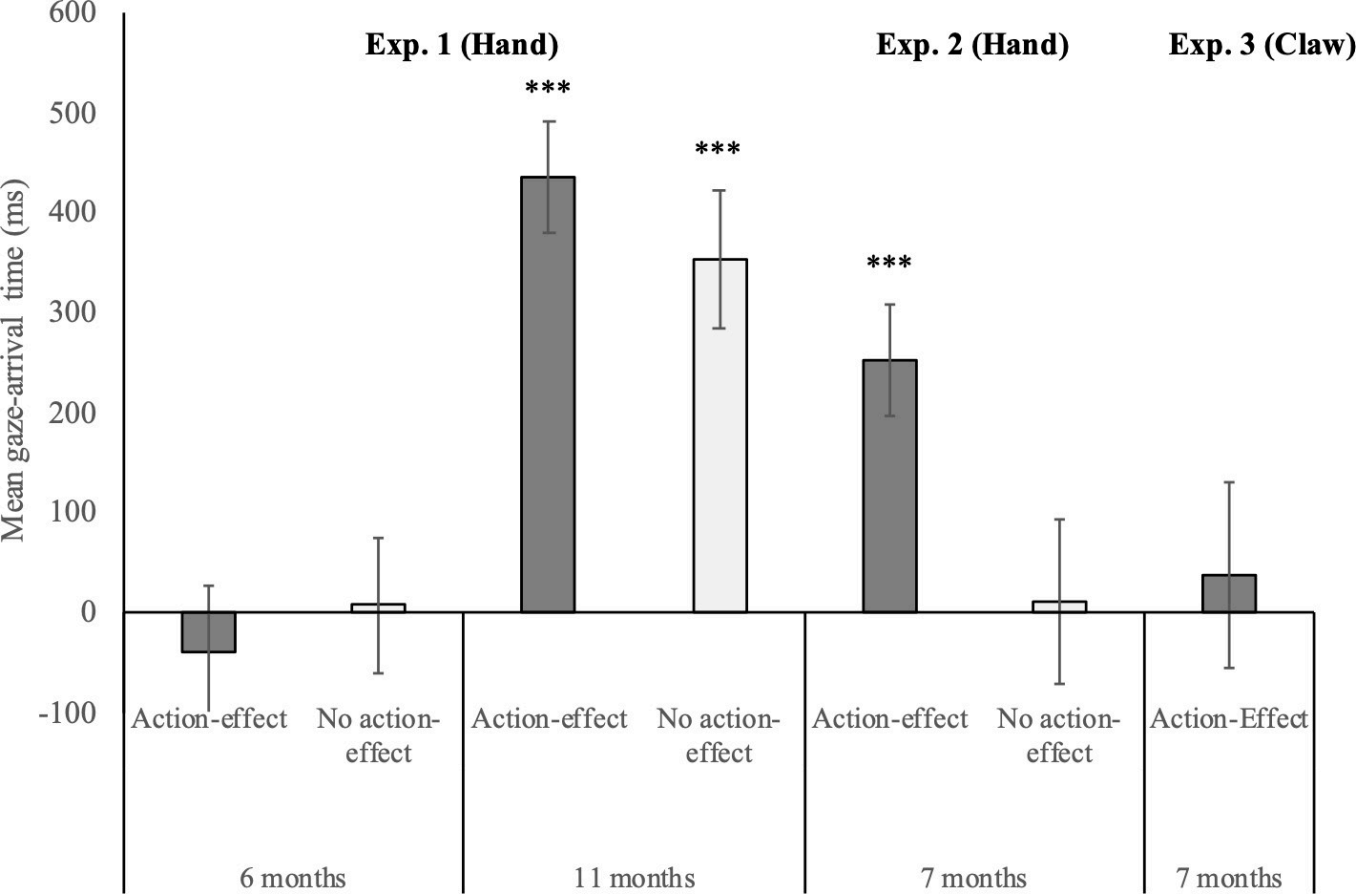
Mean gaze-arrival times for the 6-, 11-, and 7-month-olds in the human hand action-effect condition and no-action-effect condition of Experiments 1 and 2, and for the 7-month-olds in the claw-action-effect condition of Experiment 3. Positive (vs. negative) values represent gaze arrival at the goal object before (vs. after) the hand/claw arrives there. Error bars represent standard-errors, and the asterisks mark predictive gaze with gaze-arrival times significantly above 0 ms, *** = *p* < .001.

The ANOVA on mean gaze-arrival times with age group (6 months vs. 11 months) and condition (action-effect vs. no-action-effect) as between-subjects factors yielded a significant main effect of age group, *F*(1,99) = 38.1, *p* < .001, η^2^ = .28, but no significant main effect of condition, *F*(1,99) = 0.1, *p* = .79, η^2^ = .001, and no significant interaction, *F*(1,99) = 0.9, *p* = .34, η^2^ = .01 (see Fig 2). The 11-month-olds shifted their gaze to the goal object significantly faster than the 6-month-olds, and contrary to our hypothesis for the 6-month-olds but confirming our hypothesis for 11-month-olds, infants of both age groups shifted their gaze to the goal object equally fast in the action-effect condition and the no-action-effect condition.

For the critical analyses regarding the quality of infants’ gaze behavior, the one-sample *t*-tests against the value of 0 ms revealed that contrary to our hypothesis, the 6-month-olds exhibited tracking gaze behavior both in the action-effect condition, *t*(25) = -0.6, *p* = .58, *r* = .1, *BF*_01_ = 5.7, and in the no-action-effect-condition, *t*(24) = 0.1, *p* = .93, *r* = .02, *BF*_01_ = 6.5. In contrast, in line with our hypothesis, the 11-month-olds showed predictive gaze behavior both in the action-effect condition, *t*(25) = 7.8, *p* < .001, *r* = .8., and the no-action-effect condition, *t*(25) = 5.1, *p* < .001, *r* = .7.

**Fig 3:**
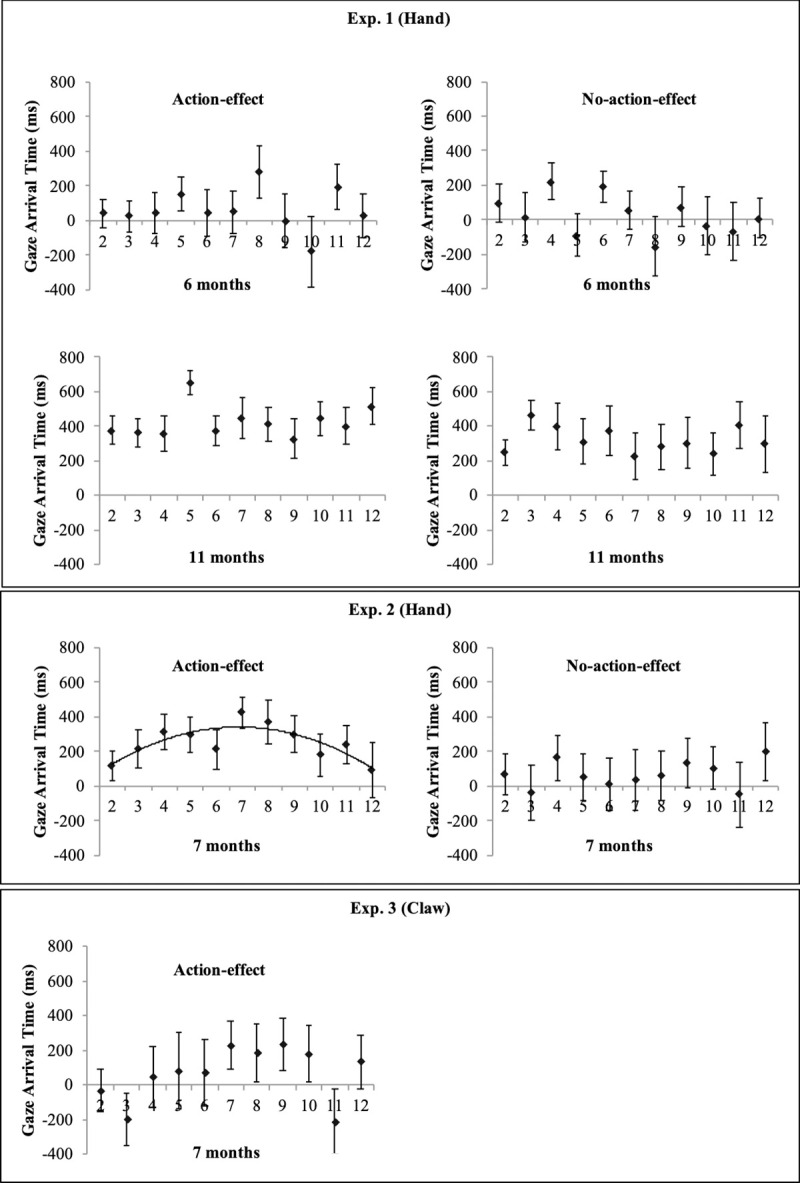
Mean gaze-arrival times across trials 2–12 for the 6-, 11-, and 7-month-olds in the human hand action-effect condition and the no-action-effect condition of Experiments 1 and 2, and for the 7-month-olds in the claw-action-effect condition of Experiment 3. Positive (vs. negative) values represent gaze arrival at the goal object before (vs. after) the hand/claw arrives there. Error bars represent standard-errors. The quadratic curve represents the regression function with most explained variance.

The regression analyses on potential learning progress in the gaze arrival times across trials 2–12 revealed no significant fit for a linear, quadratic, or logarithmic function in the two age groups and conditions, all *p*s > .22 (see Fig 3). The range of valid data points in the single trial-means for the 6-month-olds was 38% - 77% in the action-effect condition and 40% - 72% in the no-action-effect condition, and for the 11-month-olds was 73% - 92% in the action-effect condition and 53% - 84% in the no-action-effect condition. The regression analyses thus revealed that infants’ gaze behavior did not change systematically across trials, which is contrary to our hypothesis for the 6-month-olds who generally exhibited tracking gaze behavior in both conditions, and confirms our hypothesis for the 11-month-olds who generally exhibited predictive gaze behavior in both conditions.

The ANOVA on mean looking-time to the agent and to the goal object during goal approach with age group and condition as between-subjects factors revealed a significant main effect of age group, *F*(1,99) = 47.2, *p* < .001, η^2^ = .32, but no significant main effect of condition and no significant interaction, both *p*s > .10. The 11-month-olds (*M* = 2337 ms, *SD* = 602) looked longer at the agent and the goal object than the 6-month-olds (*M* = 1448 ms, *SD* = 715), and infants in both conditions attended to the agent and the goal object during goal approach approximately for the same amount of time.

### Discussion

In Experiment 1, we aimed at investigating at which age infants need to draw on the presence of an agency cue (i.e., production of a salient action effect) to visually predict the goal of an ongoing human grasping action. We examined the assumed interplay between bottom-up information such as agency cues and top-down information in the form of action knowledge [12] in experienced graspers at 11 months and in infants who still rely mainly on tactile feedback in their active grasp control at 6 months [21]. Overall, mean gaze-arrival times were higher (indicating faster gaze-shifts towards the goal object) in 11- than 6-month-olds, but in neither age group, the presence of a salient action effect influenced gaze shifts to the goal object. Regarding the quality of infants’ gaze behavior, the comparison to the threshold of 0 ms showed that only the 11-month-olds followed the expected pattern and showed predictive gaze in both conditions, indicating that the salient action effect was redundant for their action prediction [8]. Here, it is interesting to note that in previous research, 11-month-olds showed predictive gaze shifts when the grasping action of a mechanical claw produced a salient action effect, but tracking gaze for the same action without the action effect [17], indicating that action effects are only redundant for familiar actions and/or agents. Against our hypotheses, however, the 6-month-olds in the current study showed tracking gaze for the human grasping actions in both conditions, indicating that the agency cue in form of a salient action effect did not help the younger infants to activate their action knowledge, which would enable goal-predictive gaze shifts via top-down influences [12].

Regression analyses revealed no systematic change of gaze-arrival times across trials 2–12 in any of the four experimental groups, indicating that the infants’ mean gaze-arrival times stayed more or less the same across the experiment. This confirmed our hypotheses for the 11-month-olds, but not for the 6-month-olds. Furthermore, looking-time analyses did not indicate differences in attention to the initial phase of the stimuli, during which gaze-arrival times were also measured, between the action-effect condition and the no-action-effect condition.

## Experiment 2

In contrast to the findings by Ambrosini et al. [22] and Kanakogi & Itakura [6], and against our expectations, the 6-month-olds in Experiment 1 did not perform goal-predictive gaze shifts for the observed the human grasping action, regardless of the salient action effect. One possible explanation is that the 6-month-olds did not yet dispose of sufficient action knowledge about grasping in order to successfully draw on the action effect to process the goal-directedness of the presented action [8] or to enable forward modeling and action-goal prediction via top-down influences [12]. Therefore, in Experiment 2, we presented slightly older infants at 7 months with the same stimuli as in Experiment 1. Because infants change to more visually-based and prospective grasp control at around 7 months [21], observing the salient action effect in the first trials might help 7-month-olds to activate their action knowledge, enabling online prediction of the upcoming action goal via top-down influences in the subsequent trials [12]. Therefore, we expected the 7-month-olds to show predictive gaze behavior in the action-effect condition, but tracking gaze in the no-action-effect condition. Regarding a learning progress, we expected the 7-month-olds to show faster gaze shifts across trials only in the presence of a salient action effect.

### Method

#### Participants

The final sample consisted of fifty-two 7-month-olds (*M* = 7.4 months, *SD* = 0.2, range = 7.0–7.9 months; 26 girls). An additional 4 infants had to be excluded due to a lack of valid trials. As in Experiment 1, the infants were randomly assigned to the action-effect condition (*n* = 26) or the no-action-effect condition (*n* = 26).

#### Stimuli, apparatus, procedure, data handling

The stimuli, apparatus, procedure, and data handling were identical to Experiment 1. The tracking ratio of the 7-month-olds in the action-effect condition (*M* = 77%, *SD* = 16) was significantly higher than in the no-action-effect condition (*M* = 58%, *SD* = 17), *t*(50) = -4.2, *p* < .001, *r* = .5. However, the 7-month-olds contributed a comparable number of valid trials in the action-effect condition (*M* = 6.8, *SD* = 2.9) and the no-action-effect condition (*M* = 6.0, *SD* = 2.3), *t*(50) = -1.1, *p* = .29, *r* = .2.

### Results

An independent-samples *t*-test yielded that in line with our hypotheses, mean gaze-arrival times were significantly faster in the action-effect condition than in the no-action-effect condition, *t*(50) = -2.5, *p* < .05, *r* = .3 (Fig 2). Critically, the quality of the 7-month-olds’ gaze behavior was influenced by the presence of the action effect: The one-sample *t*-tests against the value of 0 ms revealed the expected predictive gaze behavior in the action-effect condition, *t*(25) = 4.5, *p* < .001, *r* = .7, and tracking gaze behavior in the no-action-effect condition, *t*(25) = 0.1, *p* = .9, *r* = .02, *BF*_01_ = 6.6.

To compare the results of Experiment 2 and of Experiment 1, we performed two additional exploratory ANOVAs. The first exploratory ANOVA on mean gaze-arrival times with age group (6-month-olds of Experiment 1 vs. 7-month-olds of Experiment 2) and condition (action-effect vs. no-action-effect) as between-subjects factors yielded a significant main effect of age group, *F*(1,99) = 4.5, *p* < .05, η^2^ = .04, no significant main effect of condition, *F*(1,99) = 2.0, *p* = .16, η^2^ = .02, and a significant interaction, *F*(1,99) = 4.3, *p* < .05, η^2^ = .04. This indicates that the 7-month-olds shifted their gaze faster to the goal object than the 6-month-olds. Additionally, and more importantly, the impact of the salient action effect differed between these two age groups. This age effect was further illustrated by a significant correlation between mean gaze-arrival times in the action-effect condition and individual age (in months and days) for the 6-month-olds of Experiment 1 and the 7-month-olds of Experiment 2, *r* = .48, *p* < .001. The correlation indicates that goal-predictive gaze does not occur abruptly, but rather progresses continuously across 5.5 and 7.9 months. The second exploratory ANOVA on mean gaze-arrival times with age group (11-month-olds of Experiment 1 vs. 7-month-olds of Experiment 2) and condition (action-effect vs. no-action-effect) as between-subjects factors again yielded a significant main effect of age group, *F*(1,100) = 15.7, *p* < .001, η^2^ = .14, but also a significant main effect of condition, *F*(1,100) = 6.0, *p* < .05, η^2^ = .06, and no significant interaction, *F*(1,100) = 1.5, *p* = .23, η^2^ = .01. The 11-month-olds shifted their gaze to the goal object significantly earlier than the 7-month-olds, and across both age groups, infants shifted their gaze to the goal object faster in the action-effect condition than in the no-action-effect condition. This direction of the condition difference was visible in the 11-month-olds’ data of Experiment 1, but apparently, the effect size was not large enough to yield a significant condition difference in the analysis of Experiment 1.

The regression analyses revealed a significant fit for a quadratic function to the 7-month-olds’ gaze-arrival times across trials 2–12 in the action-effect condition (y = 30.2+106.2x-9.0x^2^, *R*^2^adj = .59, *F*(2,8) = 8.2, *p* < .05. The range of valid data points in the single trial-means was 42% - 81% in the action-effect condition and 34% - 80% in the no-action-effect condition. The quadratic trend indicates that the mean gaze-arrival times in the action-effect condition became faster across the first half of the experiment (until trial 7), but then slowed down towards the end. Thus, the quadratic function indicates that it seemed to be difficult for the 7-month-olds to maintain predictive gaze behavior over the 12 trials. For the no-action-effect condition, there was no significant fit for a linear, quadratic, or logarithmic function, all *p*s > .45, indicating no systematic change across trials in the tracking gaze behavior that was generally exhibited by this group of 7-month-olds.

Finally, an independent samples *t*-test revealed no significant differences in 7-month-olds’ mean looking-time to the agent and to the goal object during goal approach between the action-effect condition (*M* = 1845 ms, *SD* = 661) and the no-action-effect condition (*M* = 1609 ms, *SD* = 806), *t*(50) = -1.2, *p* = .25, *r* = .2.

It needs to be noted that the age range of the 6-month-olds’ in Experiment 1 (5.5–6.9 months) was wider than that of the 7-month-olds’ in Experiment 2 (7.0–7.9 months). To ensure that this difference did not significantly impact the mean gaze-arrival times, we split the 6-month-olds into a group of *n* = 18 infants aged 5.5–5.9 months and a group of *n* = 33 infants aged 6.0–6.9 months. We then performed an ANOVA on the 6-month-olds’ mean gaze-arrival times with subgroup and condition as between-subjects factors and found no significant main effects or interaction, all *p*s > .25. This indicates that the relatively wider age range of the 6-month-olds did not have a systematic impact on our main variable.

### Discussion

Experiment 2 revealed that infants at 7 months of age, who dispose of more action knowledge than 6-month-olds [21], benefited from the addition of a salient action effect when observing a human manual grasping action. The 7-month-olds showed the expected pattern of predictive gaze behavior in the action-effect condition, but tracking gaze behavior in the no-action-effect condition. Additional exploratory analyses confirmed these findings by revealing that the addition of a salient action effect impacted the 7-month-olds’ gaze behavior in Experiment 2, but not the 6-month-olds’ in Experiment 1. However, a comparison between the 7-month-olds of Experiment 2 and the 11-month-olds of Experiment 1 showed that both age groups were able to draw on the action effect to inform their predictions, but only the 11-month-olds were able to systematically predict the hand’s action goal regardless of the action effect. This provides first evidence for the proposed interplay between bottom-up and top-down information [12] by showing that from 7 months onwards, but not at 6 months of age, infants draw on salient action effects to visually predict the goal or target location of an ongoing human grasping action. Furthermore, as expected, the 7-month-olds’ mean gaze-arrival times in the action-effect condition showed systematic changes across the course of the experiment, with fast learning progress across the first trials, but decreasing gaze-arrival times in the final trials, which indicates the 7-month-olds’ difficulty to maintain goal-predictive gaze behavior across the 12 trials. Finally, the 7-month-olds did not show differing looking-times to the agent and the goal object between conditions, suggesting that there were no differences in infants’ attention during the initial phase of the stimuli between conditions. This rules out the possibility that mere attentional mechanisms, triggered by the presentation of the action effect in only one of the conditions, might account for the condition difference in the 7-month-olds’ mean gaze-arrival times, which were also measured during goal approach, while the stimuli were still perceptually identical in both conditions.

However, compared to the 6-month-olds of Experiment 1, the 7-month-olds of Experiment 2 probably did not only have richer knowledge about grasping actions, but also disposed of more mature attentional and memory capabilities in general. Therefore, to test this alternative explanation that the 7-month-olds’ ability to predict the action goal in the presence of a salient action effect in Experiment 2 was due to generally more advanced cognitive abilities through brain maturation rather than specific for action processing and based on more advanced action experience, we presented 7-month-olds in Experiment 3 with similar stimuli as in the action effect-condition of Experiments 1 and 2, but with a mechanical claw as the grasping agent.

## Experiment 3

In previous work, 6- and 7-month-olds did not display goal-predictive gaze shifts for a grasping mechanical claw [6], which was taken as support for the experience-based accounts [3]. Further, the assumed interplay of bottom-up processes (i.e., perceptual analyses of visual features of the agent and the beginning of the movement) and top-down influences (exerted by activation of stored action representations) should not work when infants have only poor action experience with the unfamiliar agent [12]. Hence, 7-month-olds should not be able to predict the goal of a grasping mechanical claw, even when it produces a salient action effect. Furthermore, the 7-month-olds should not show faster gaze shifts across trials for the mechanical claw, despite the presence of the salient action effect. This pattern of results would rule out that the 7-month-olds, but not the 6-month-olds, in the action-effect conditions of Experiments 1 and 2 predicted the goal of the ongoing human grasping action only because the 7-month-olds used their more advanced cognitive abilities for memorizing the upcoming action effect, with no further action processing involved.

### Method

#### Participants

The final sample consisted of twenty-four 7-month-olds (*M* = 7.5 months, *SD* = 0.3, range = 7.0–7.9 months; 12 girls) in the claw-action-effect condition. An additional 8 infants had to be excluded due to a lack of valid trials.

#### Stimuli, apparatus, procedure and data handling

The stimuli, apparatus, procedure, and data handling were identical to Experiment 1, with the following exceptions: In the grasping videos, the human hand was replaced by a mechanical claw (see Fig 1). The timing of the claw and hand videos was identical, except for slight differences in the durations of the lifting/action effect (claw: approx. 2600 ms; hand: approx. 2800 ms), of the still frame (claw: approx. 2300 ms; hand: approx. 2400 ms), and the total duration (claw: approx. 8400 ms; hand: approx. 8700 ms). The tracking ratio was *M* = 74%, *SD* = 16, which was comparable to the 7-month-olds in the action-effect condition of Experiment 2, *t*(48) = -0.53, *p* = .60, *r* = .08, and was significantly higher than in the no-action-effect condition of Experiment 2, *t*(48) = -3.6, *p* < .01, *r* = .5. However, the 7-month-olds of Experiment 3 contributed an average number of *M* = 6.1 valid trials (*SD* = 3.2), which was comparable to the action-effect condition, *t*(48) = -0.84, *p* = .40, *r* = .1, as well as to the no-action-effect condition, *t*(48) = -0.06, *p* = .96, *r* = .01, of Experiment 2.

### Results and discussion

The one-sample *t*-tests against the value of 0 ms revealed that as expected, the 7-month-olds showed tracking gaze in the claw-action-effect condition (*M* = 36.1, *SE* = 92.3), *t*(23) = 0.39, *p* = .7, *r* = .08, *BF*_01_ = 5.9. Furthermore, the regression analyses revealed no significant fit for a linear, quadratic, or logarithmic function across trials 2–12, all *p*s > .36. The range of valid data points in the single trial-means was 41%– 79% for the 7-month-olds in the claw-action-effect condition. Thus, there was no systematic change across trials in the generally exhibited tracking gaze behavior.

To compare the results of Experiment 3 with the previous findings, we again performed additional exploratory analyses. We compared the (tracking) mean gaze-arrival times from the 7-month-olds in the claw-action-effect condition of Experiment 3 with the (predictive) mean gaze-arrival times from the 7-month-olds in the action-effect condition of Experiment 2. To account for a violation of homogeneity of variance between the groups, we conducted a Mann-Whitney-U test, which yielded a significant difference in mean gaze-arrival times between the two groups, *U* = 208, *p* < .05. This suggests that the 7-month-olds reacted differently to the grasping action performed by the human versus the mechanical agent and that the predictive gaze shifts in Experiment 2 were not just driven by the 7-month-olds’ more advanced cognitive abilities or the presence of the salient action effect.

This was also supported by a comparison of the 7-month-olds’ mean looking-times to the agent and to the goal object during goal approach. An independent-samples *t*-test yielded no significant difference in mean looking-time between the hand-action-effect condition of Experiment 2 (*M* = 1845 ms, *SD* = 661) and the claw-action-effect condition of Experiment 3 (*M* = 1654 ms, *SD* = 727), *t*(48) = 0.97, *p* = .34, *r* = .1. Further, in order to control whether the claw in Experiment 3 was more interesting to the 7-month-olds compared to the hand in Experiment 2, which might hinder gaze shifts to the goal object, we compared mean looking-times only to the agent during goal approach in Experiment 2 (hand-action-effect) and Experiment 3 (claw-action-effect). An independent-samples *t*-test again yielded no significant difference, *t*(48) = 1.8, *p* = .09, *r* = .3, and numerically, looking-times were longer to the hand (*M* = 703 ms, *SD* = 342) than to the claw (*M* = 537 ms, *SD* = 327). This finding rules out the alternative explanation that the attractiveness of the claw has prevented the 7-month-olds from predictively shifting their gaze from the agent to the goal object, and it indicates that the hand and the claw were equally attractive to watch for the 7-month-olds.

## General discussion

The analyses of Experiment 1 revealed that first, the 11-month-olds shifted their gaze to the goal object significantly faster than the 6-month-olds, which is in line with other eye tracking studies that found faster gaze shifts to a goal object with increasing age [1, 6]. Second, infants in both age groups shifted their gaze to the goal object about equally fast in both conditions, suggesting that neither age group drew on the action-effect during action observation. Further, and most importantly, our analyses of the quality of infants’ gaze behavior indicated different underlying mechanisms for the lacking impact of the presence or absence of the salient action effect in the two age groups: As expected, the 11-month-olds showed predictive gaze behavior regardless of the action effect. This is in line with the assumption that 11-month-olds already had sufficient top-down action-knowledge about grasping actions, which in turn rendered the visual availability of the agency cue as bottom-up information redundant [8, 11, 12].

However, against our expectations, the 6-month-olds showed tracking gaze behavior in both the action-effect and the no-action-effect condition. This suggests that at 6 months of age, infants still fail to draw on a single agency cue in the form of a salient action effect to visually predict the goal of an ongoing human grasping action. These results fit to a body of work reporting that 6-month-olds are not able to predict the goal of contralateral reaching actions that are still relatively new to them [24] or to predict the goal of feeding actions, especially when their manual proficiency was low [23]. On the other hand, several previous studies have found goal-predictive gaze behavior in 6-month-olds for human grasping actions that did not produce salient action effects [6, 22]. Compared to our study, however, the stimuli used in these studies provided several additional cues that could have helped the infants in predicting an action effect that could happen after the goal grasp. For example, in both studies the infants saw an agent performing different kinds of actions, like grasping, back of hand, full grasp, or pincer grasp, being performed alternately on one of two objects in a within-subjects design. Therefore, the infants saw the agent acting in different ways to approach the goal object, which could be interpreted as equifinality of goal achievement [8], and this additional agency cue possibly made the task easier for the infants. Furthermore, Kanakogi and Itakura [6] presented the stimuli from an egocentric point of view, which might have enabled infants to relate the observed actions to their own experience.

We take our results to indicate that the 6-month-olds did not yet dispose of sufficient action knowledge about grasping actions and the effects they elicit to use either their knowledge or a single agency cue for goal predictions [12, 17, 18]. In the looking-time study by Bíró and Leslie [8], 6-month-olds needed more agency cues during action observation than older infants to detect a (mis)match between an expected and the actual goal of a completely observed goal-directed action. Thus, it is possible that when more bottom-up information in the form of agency cues were present, even 6-month-olds could perform predictive gaze behavior for an ongoing human grasping action.

A critical finding of our study is that the 7-month-olds in Experiment 2 were able to draw on the agency cue in order to predict the goal of the grasping human hand: The mean gaze-arrival times of the 7-month-olds differed significantly in the two conditions of Experiment 2, with the expected pattern of predictive gaze behavior in the action-effect condition, but tracking gaze behavior in the no-action-effect condition. Exploratory comparisons between the results of the 6-month-olds of Experiment 1 and the 7-month-olds of Experiment 2 also revealed that indeed the 7-month-olds’ gaze behavior changed in relation to the presence or absence of a salient action effect, whereas the 6-month-olds’ gaze behavior did not. Finally, a comparison between the 11-month-olds of Experiment 1 and the 7-month-olds of Experiment 2 showed that both age groups on average showed faster predictive gaze shifts in the action-effect condition compared to the no-action-effect condition. Here, the analyses of the quality of infants’ gaze revealed that only the 7-month-olds relied on the action effect, with predictive gaze in the action-effect condition, but tracking gaze in the no-action-effect condition. The 11-month-olds, in contrast, showed predictive gaze behavior regardless of the action effect.

Thus, the results of Experiment 1 and 2 support the idea of an inverse relation between an observer’s top-down action knowledge and their need to draw on available bottom-up information in the form of agency cues to process the goal-directedness of an observed action [8, 11, 12]: At 6 months of age, the infants’ stored action representations are still weak and cannot be activated based on the presentation of a human hand producing a salient action effect. At 7 months of age, however, the infants’ stored action representations are stronger and can successfully be activated by the observation of the human hand performing a salient action effect, leading to goal predictions in the action-effect condition. Finally, at 11 months of age, the infants’ stored action representations are strong enough to be activated by mere observation of a hand approaching and touching a goal object, rendering the additional action effect irrelevant for successful predictive gaze behavior. When we compare the results from the 11-month-olds to previous research with adults, there does not seem to be much difference when it comes to their propensity to predict the goal of human grasping actions, even in the absence of any additional behavioral cues [2, 6]. However, previous research [6] suggests that adults would show faster gaze-shifts than the 11-month-olds in our study, based on adults’ richer knowledge about and experience with preforming and observing grasping actions. Adults also have a more developed oculomotor system and might learn faster from simple stimuli. Still, the predictive gaze patterns found in our study suggest that at the end of the first year of life–at least in the context of simple human grasping actions–infants’ predictive gaze behavior is quite similar to the “mature” gaze behavior of adults.

It is important to note that, compared to the 6-month-olds, the 7-month-olds did not only dispose of more knowledge about grasping actions, but they also had more matured general cognitive abilities. The process of goal prediction needs to be performed during only a few hundred milliseconds and is highly constrained by stimulus timing [25, 26], which makes goal-predictive gaze shifts difficult to achieve for young infants. However, the results of Experiment 3 devaluated the explanation that the 7-month-olds in the human-action-effect condition of Experiment 2 produced predictive gaze behavior simply because they were able to memorize the action effect and/or merely formed a visual association between the movement and the subsequent action effect. Crucially, the 7-month-olds did not show predictive gaze behavior when non-human grasping (by a mechanical claw) was presented with only a single agency cue. Further, an exploratory comparison between the hand-action-effect condition of Experiment 2 and the claw-action-effect condition of Experiment 3 suggested that the 7-month-olds reacted differently to the salient action effect, depending on whether the agent was human or mechanical. This is in line with the looking-time findings by Bíró and Leslie [8] that infants around 6 months of age did not attribute goal-directedness to a non-human agent unless it displayed a combination of several agency cues. When we consider only younger infants’ limited cognitive abilities, it seems paradox that adding various agency cues, which definitely increases cognitive load during action observation, supports (rather than hinders) younger infants’ action-goal prediction. However, the beneficial influence results only when this additional bottom-up information can be combined with stored knowledge about a familiar action, confirming the assumed interplay between bottom-up and top-down processes in infant action-goal prediction [12, 27]. Additionally, our analyses ruled out an alternative explanation for the 7-month-olds’ tracking gaze in the claw-action-effect condition. The 7-month-olds’ looking-times during goal approach were similar for to the hand in Experiment 2 and the claw in Experiment 3 (and numerically, looking-times were longer to the hand than to the claw), suggesting that the differences in predictive gaze behavior cannot be explained by differences in infants’ attention to the (un-)familiar agent.

Regarding the 11-month-olds’ predictive gaze in the no-action-effect condition, our data cannot reveal whether this relies on the older infants’ greater grasping experience or on their generally more mature cognitive abilities. In training studies, already 3-month-olds showed goal-related looking-times that indicate the attribution of goal-directedness to human grasping actions, but only after having gathered agentive experience with picking off objects with ‘sticky mittens’ [28–30]. In a similar vein, it would be interesting to examine whether and at which age a grasping training prior to video presentation would influence infants’ subsequent goal-predictive gaze behavior. This could be a further step to disentangle the influence of cognitive maturation and growing action experience on infants’ goal prediction for different actions and/or agents.

It is important to note that our findings do not favor an experience-based account over a cue-based account or vice versa, but rather suggest that agency cues interact with stored action knowledge to enable goal prediction for ongoing actions [12]. Interestingly, although all infants were at an age where they were fully capable of performing grasping actions themselves, predictive gaze behavior did not occur in the no-action-effect condition of the 7-month-olds or in either condition of the 6-month-olds. These results do not contradict the notion that goal prediction might result from direct activation of the motor system through the mirror-neuron system [2, 3, 31], but they question the idea that motor experience alone acts as a necessary and sufficient factor in this context [11]. The results rather suggest that agency cues, such as salient action effects, or action knowledge help an observer to identify at which goal an action is directed [13], and it has been proposed that this goal identification then elicits predictive gaze behavior through motor simulation, given that the latter can be based on action experience [11].

Regression analyses revealed that the 7-month-olds in the action-effect condition of Experiment 2 showed increasing mean gaze-arrival times across the first half of the experiment, and decreasing mean gaze-arrival times across the second half. On the one hand, this quadratic function is different from other studies reporting a logarithmic function with increasing gaze-arrival times across the experiment for 11-month-olds when observing human grasping actions [5, 19]. Thus, the data of the 7-month-olds in the action effect-condition only partly (i.e., in the first half of the experiment) confirmed our expectation that seeing the action effect would activate infants’ (increased, but probably still weak) stored action knowledge. On the other hand, because our infants were younger than the ones in previous studies, it’s reasonable to assume that although the 7-month-olds initially were able to learn the association between the agent, the movement, and the action effect, their ability to maintain goal-predictive gaze behavior, which requires cognitive effort [25, 26], decreased in the second half. Furthermore, previous learning effects for gaze-arrival times across trials were reported to be relatively unstable across conditions [5, 19] and therefore seem to be based on processes not yet completely understood. Similarly, the present learning data suggest a complex interaction of different factors, for example the observers’ age and action experience, motivation and attention, availability of agency cues [18], features of the agent and the goal such as saliency [19], as well as still unknown variables that need to be systematically investigated in future studies.

It is worth noting that due to the fact that the stimuli only consisted of an agent approaching and acting on one goal object, our results do not reveal whether the infants actually predicted the action goal or the end location of the movement [32]. Rather, our results grant insights into whether the presence of a salient action effect enabled the infants to show goal-predictive gaze behavior in general, as a first step to investigate crucial aspects in this developing ability, such as the interplay between agency cues and action knowledge [12]. Building on this, future research could aim at further investigating how agency cues and action knowledge influence affect different aspects of goal-predictive gaze behavior by also adopting stimuli in which more than one goal object is presented.

Taken together, our results suggest that first, agency cues such as a salient action effect play an important role for 7-month-olds’, but not 6- or 11-month-olds’, goal predictions when observing human goal-directed grasping actions. Second, the 7-month-olds’ ability to draw on the action effect for goal prediction does not seem to rely on the infants’ comparatively advanced cognitive abilities, because the impact of the action effect found in Experiment 2 did not generalize to the same action carried out by a mechanical claw in Experiment 3. Thus, our results provide first evidence for an interplay between top-down action knowledge and the need to draw on agency cues as bottom-up information in order for infants to successfully predict the goal or end state of an ongoing simple goal-directed action [8, 11, 12, 27]. Future studies could build on these findings and further investigate how infants’ need to draw on agency cues during action observation interacts with their action knowledge. For example, the 7-month-olds in Experiment 2 showed strikingly similar gaze behavior when observing a human hand compared to the gaze behavior of 11-month-olds when observing similar actions of a mechanical claw [17, 18]. Therefore, it might be interesting to investigate whether the presence of a salient action effect would result in a similar developmental course of infants’ tracking and predictive gaze behavior in the context of non-human agents, albeit at a later age range. This would shed further light on the ongoing debate about how goal-predictive gaze behavior develops across the first years of life and on which crucial factors are involved in this process.

## Supporting information

S1 Fig Box-plot of the mean gaze-arrival times for the 6-, 11-, and 7-month-olds in the human hand action-effect condition and no-action-effect condition of Experiments 1 and 2, and for the 7-month-olds in the claw-action-effect condition of Experiment 3. (TIF)

S1 File Data sheet with all relevant data from Experiments 1–3. (XLSX)
