# Supplementary figures and images for: The impact of salient action effects on 6-, 7-, and 11-month-olds’ goal-predictive gaze shifts for a human grasping action
Source: PLoS One. 2020 Oct 2;15(10):e0240165. doi: 10.1371/journal.pone.0240165 (PMC7531859; doi:10.1371/journal.pone.0240165)

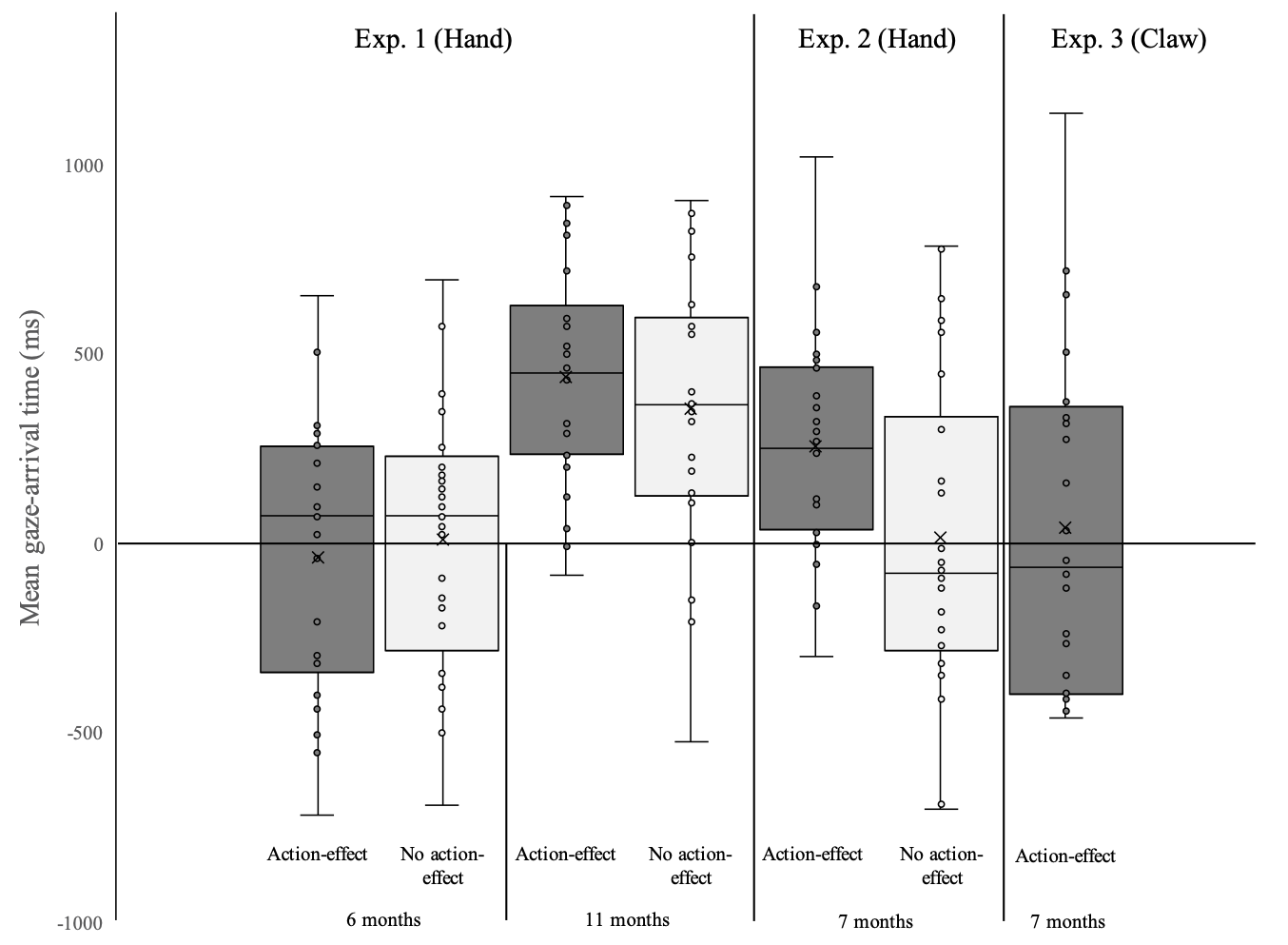

Supplement: S1 Fig — (TIF) [file pone.0240165.s001.tif]
